# Microplastics in the marine environment of St. Mary's Island: implications for human health and conservation

**DOI:** 10.1007/s10661-023-11651-6

**Published:** 2023-08-12

**Authors:** Rizwan Khaleel, Gokul Valsan, Nelson Rangel-Buitrago, Anish Kumar Warrier

**Affiliations:** 1 Department of Sciences, Manipal Institute of Technology, Manipal Academy of Higher Education, Manipal, Karnataka 576104 India; 2 Department of Civil Engineering, Manipal Institute of Technology, Manipal Academy of Higher Education, Manipal, Karnataka 576104 India; 3 Programa de Física, Facultad de Ciencias Básicas, Universidad del Atlántico, Puerto Colombia, Atlántico, Colombia; 4 Centre for Climate Studies, Manipal Academy of Higher Education, Manipal, Karnataka 576104 India

**Keywords:** Microplastics, St. Mary’s Island, Polymer Hazard Index, Potential Ecological Risk Index, Marine pollution

## Abstract

Microplastics have now been identified as a class of emerging pollutants and is considered as a threat to aquatic organisms. This baseline paper investigated the distribution, composition, and potential ecological risks of microplastic (MP) pollution on St. Mary's Island, revealing an average abundance of 0.218 particles/L in water samples. Blue fibres and white foams were the primary MPs identified, and fishing activities and packaging were the main sources of pollution. Six types of polymers were identified: low-density polyethylene (LDPE), polystyrene (PS), polyamide (PA), polypropylene (PP), polyethylene (PE), and high-density polyethylene (HDPE). The Polymer Hazard Index (PHI) and Potential Ecological Risk Index (PERI) indicated a medium environmental risk for the island. Additionally, it was discovered that MPs’ surfaces contained dangerous substances that could endanger aquatic life. The research emphasizes the significance of implementing measures such as responsible disposal, management, elimination, regulatory policies, and local administration techniques to mitigate the impact of MP pollution on the island’s shores and marine biota. This research provides a baseline for monitoring MP contamination and underscores the need for continuous investigation to assess their impacts on marine life.

**Supplementary Information:**

The online version contains supplementary material available at 10.1007/s10661-023-11651-6.

## Introduction

The excessive use of plastics, without considering their long-lasting effects, is known to harm our planet. In the 15 years since 2000, plastic production has increased by a staggering 70%. Notably, 39.7% of this increase can be attributed to the packaging industry (Kosior & Mitchell, 2020). In recent years, global plastic production has continued to rise, increasing from approximately 359 million metric tonnes to 390.7 million tonnes in 2021 (Plastics Europe, 2022). On average, approximately 14 million metric tons of plastic waste reach the global oceans each year (IUCN, 2018).

Plastic waste generated on land is carried into the ocean and persists there for extended periods (Li et al., 2016), eventually breaking down into tiny pieces of plastic. The term “microplastics” (MPs) refers to these tiny fragments of plastic, which range in size from 1 µm to 5 mm. (Amrutha et al., 2023; Thompson et al., 2004). They can also be divided into primary and secondary MPs. Primary MPs are microbeads that are used in facial cleansing products or in industrial processes, and in synthetic fabrics (Andrady, 2011). Secondary MPs, on the other hand, are the broken-down remains of larger plastic debris. The breakdown of these materials is accelerated by various physical, chemical, and biological processes, including exposure to ultraviolet radiation, the mechanical action of waves, abrasion from coarse rock particles, and the formation of biofilms (Kernchen et al., 2022).

Carpenter et al. (1972) was the first to observe small pieces of plastic floating on the water’s surface off the coast of New England. Since then, numerous studies have discovered MPs in locations such as the Arctic (Choudhary et al., 2022; Morgana et al., 2018), the Antarctic (Jones-Williams et al., 2020), and even the deepest parts of our oceans (Zhang et al., 2020). Rivers transport pollutants from land to the oceans (Amrutha & Warrier, 2020). Upon entering the oceans, waves and currents play a significant role in dispersing MPs into offshore areas. Additionally, various marine organisms, including fish, molluscs, crustaceans, and seabirds, ingest these MPs (Amélineau et al., 2016; da Costa et al., 2023; Hamilton et al., 2021). MPs can adsorb pollutants from both terrestrial and aquatic environments, including heavy metals, persistent organic pollutants (POPs) as well as host the colonies of bacteria that carry antibiotic genes (Keswani et al., 2016; Liu et al., 2021). Additional biochemical stress can be brought on by toxic substances like phthalates and bisphenols that can enter the organisms.

In India, MPs have been discovered in lakes, rivers, estuaries, soils, and various living organisms (Amrutha & Warrier, 2020; Pandey et al., 2021; Sruthy & Ramasamy, 2017; Unnikrishnan et al., 2023; Valsan et al., 2023; Warrier et al., 2022). Islands are critical ecosystems, providing habitats for a diverse range of species (Kueffer et al., 2016; Royle, 2008). They hold cultural importance, as they support the livelihoods of local inhabitants (Gillespie & Clague, 2009; Royle, 2014). However, islands are often threatened by both natural and human-induced factors, such as increased tourism, overexploitation of natural resources, and rising sea levels due to climate change (Baldacchino & Niles, 2011; Rangel-Buitrago et al., 2019; Royle, 2014; Walker & Bellingham, 2011).

Despite their significance, there are limited reports on the presence and distribution of MPs in the Indian Ocean islands, particularly the Arabian Sea (Khaleel et al., 2022; Mugilarasan et al., 2017). To close this knowledge deficit, we investigated the spatial distribution of tiny plastic particles in water samples collected from St. Mary’s Island located in the southeast Arabian Sea. Our goals were to: (i) analyze the abundance, shape, size, and colour of microplastics; (ii) determine the polymer composition of MPs and look at their surface morphology; and (iii) determine the possible risks that MPs pose to the local biota.

## Methods and materials

### Study area and water sampling

**Fig. 1:**
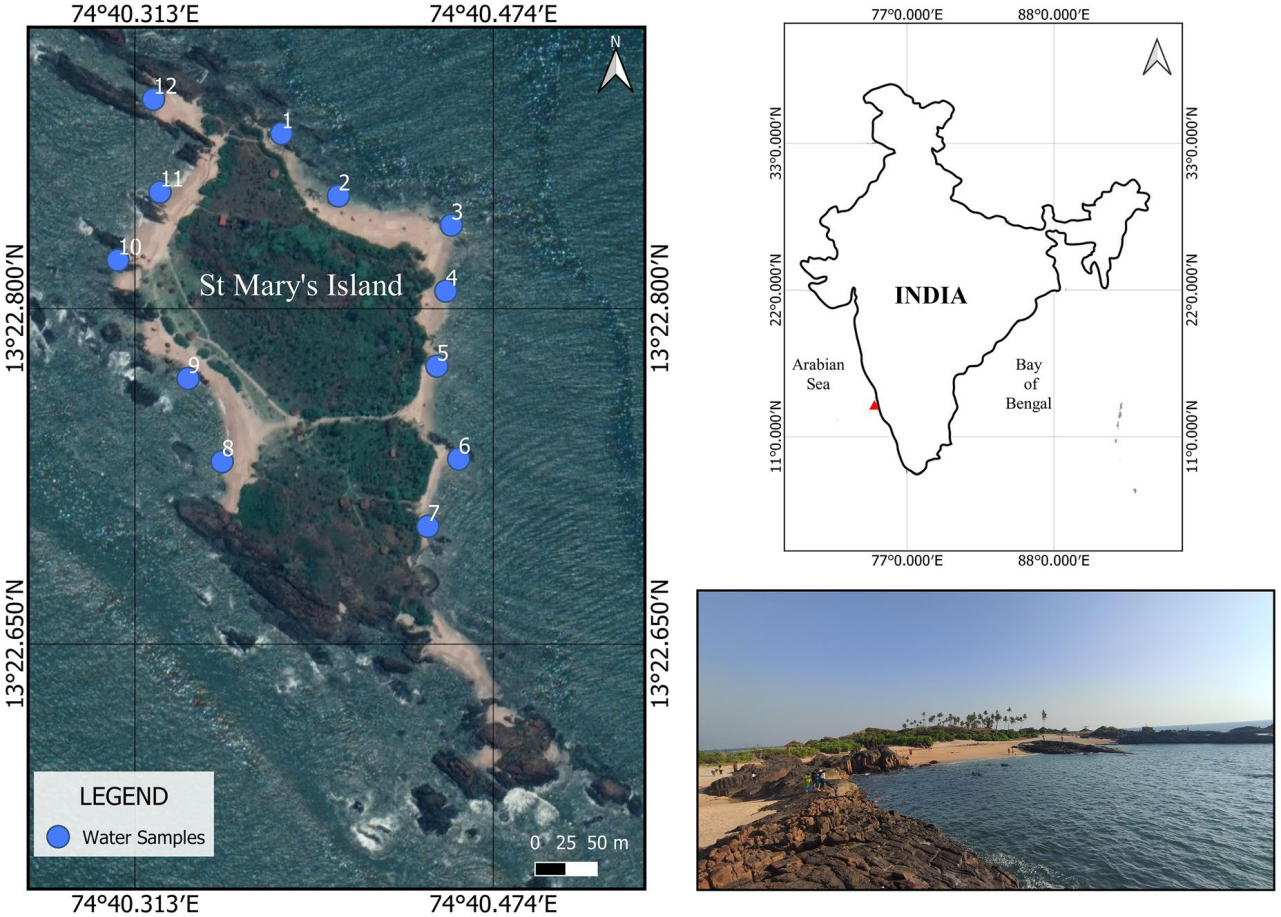
The location of St Mary’s Island in the southeastern Arabian Sea

St. Mary’s Island, also known as Coconut Island or Parashurama Dveepa, is in the Udupi Region (Fig. 1). The island is three kilometres off the shore of Malpe in Karnataka, southwest India, at 13° 22′ 46.2′′ N and 74° 40′ 22.8′′ E. It has a 2.17-km circumference and a 0.11 square kilometre surface size. Beach cast can build up on shorelines and other places at elevations of 12 m or less (Selvam et al., 2011). Coconut Island, Bahudurgari Island, North Island, and South Island together form the group of four islands that comprise St. Mary’s Island. These islands are not perfectly aligned in a north–south orientation (Selvam et al., 2011). The study area serves as an excellent example of a volcanic island formed by rift extensional movements (Marion hotspot – Melluso et al., 2009). Pyroxene rhyolites, exposed in the island’s well-known columnar joints, underwent Au-Pb-Zircon dating, which estimated their age to be 91.2 ± 0.2 million years (Late Cretaceous; Bhushan et al., 2010; Torsvik et al., 2000).

Most of the year in the archipelago is associated with tropical weather, and different wind patterns throughout the year cause a monsoon season between June and September. This results in 2,893 mm of precipitation on average each year, and the average yearly temperature is 26.5 °C. The island is affected by strong gusts during the southwest monsoon. During the northeast monsoon, however, winds blow from the north (Udupi District Environmental Plan, accessed on May 24, 2022). In 2016, the Geological Survey of India designated St. Mary's Island as a National Geological Monument (Geo-Heritage Sites; https://pib.gov.in/newsite/printrelease.aspx?relid=137573, accessed on 11th March 2023). To promote sustainable use of the island, a time limit of one hour has been set for each visitor to engage in tourist activities. This measure aims to manage the number of tourists visiting the island, which has become a popular destination for both domestic and international visitors. Access to the island is limited to boats operating from October to May each year.

A total of 12 surface water samples, were collected from various locations on St. Mary's Island on January 22, 2022, using a stainless-steel bucket (Fig. 1). A stainless-steel test sieve with a mesh size of 0.1 mm (Haver Scientific) was used to filter about 100 L of water. The filtered residue was carefully transferred to a stainless-steel container. The samples were then brought to the laboratory and kept there until analysis at 4 °C.

### Extraction of microplastics

The isolation of MPs from the water samples was performed using a modified NOAA (National Oceanic and Atmospheric Administration) method (Masura et al., 2015; Rodrigues et al., 2018). The water samples were filtered through a series of stainless-steel sieves stacked on top of each other in decreasing mesh size (5 mm, 1 mm, 0.3 mm, and 0.1 mm). Particles larger than 5 mm were kept separately, while those between 5 mm and 0.1 mm were transferred into separate glass beakers and oven-dried at 50 °C for 24 h.

The samples underwent wet peroxide oxidation (WPO) treatment to digest organic compounds (Masura et al., 2015). The desiccated sample was mixed with 20 ml of an aqueous 0.05 M Fe (II) solution and 30 ml of 30% H_2_O_2_ before being heated on a hot plate at 50 °C. The Fe (II) Solution serves as a catalyst to accelerate the organic matter digestion process when combined with 30% H_2_O_2_. To isolate the floating MPs from the denser inorganic material, 150 ml of ZnCl_2_ (933.3 g L^−1^ with a density of 1.6 g cm^−3^) was added to the samples enabling the separation of heavier organic matter settling at the bottom from lighter MP particles floating in the solution. The lighter particles were separated according to their size after the denser ones were removed. The gathered particles were put into petri dishes and dried for 24 h in a hot-air oven at 50 °C.

### Identification of microplastics

The presence of microplastics was observed using a Nikon SMZ745 Stereozoom Microscope with a magnification of 40x. The identified MPs were picked up using stainless steel forceps and transferred into glass vials. A hot-needle test was conducted to identify problematic particles (Ruggero et al., 2020). Based on their shape, the MPs were categorized into fibre, foam, pellet, fragment, and film (Ruggero et al., 2020).

Fourier Transform Infrared Spectroscopy (FTIR) with Attenuated Total Reflectance (ATR) was used to analyse the polymer composition of the identified MPs (Browne et al., 2011). The obtained spectra were compared with Open Specy, an online reference spectra library, to ensure accuracy and validity (Cowger et al., 2021). Around 20 samples belonging to the size range of 1–5 mm were tested for their polymer composition using ATR-FTIR. A Scanning Electron Microscope (EVO MA18 with Oxford EDS(X-act)) was utilized to investigate the surface characteristics of the selected microplastics (MPs) larger than 1 mm. The microscope has a magnification range of 1x (minimum) to 100,000x (maximum). Since plastics are non-conductive materials, gold sputtering is used to enhance the conductivity of plastic samples, facilitating their imaging using a scanning electron microscope. Energy-dispersive X-ray spectroscopy (EDS) was used to obtain real-time compositional spectra of microplastics (Wang et al., 2017).

### Risk assessment studies

Risk assessment indices of three types were calculated to study the extent of microplastic pollution in the study area. The Polymer Hazard Index (PHI) was computed by recognizing their chemical toxicities. Apart from the abundance of microplastics (MPs), the toxicity of MPs is primarily influenced by the type of polymer they are made of (Qiu et al., 2023). The formula used to calculate PHI is:

$$\mathrm{PHI}=\sum {S}_{n} \times {P}_{n}$$

where, *S*_*n*_ is the hazard score of polymers and *P*_*n*_ is the proportion of each MPs polymer type. The PHI is calculated using *S*_*n*_, the hazard score of polymers, and *P*_*n*_, the proportion of each MP's polymer type. The PHI was classified into different hazard levels as described by Lithner et al. (2011). The five hazard levels of PHI are as follows: 0–1 (I), 1–10 (II), 10–100 (III), 100–1000 (IV), and > 1000 (V) (Qiu et al., 2023; Nithin et al., 2022, 2023; Liu et al., 2021; Ranjani et al., 2021; Li et al., 2020a, b).

The second index, Potential Ecological Risk Index (PERI), was proposed by Hakanson (1980) to evaluate the potential risk to the ecology of a given environment. This index helps assess the combined effect of diverse pollutants contaminating the environment (Peng et al., 2018). The PERI was calculated using the following equations:

$${C}_{f}^{i}=\frac{{c}^{i}}{{c}_{n}^{i}}$$

$${T}_{r}^{i}=\sum_{n=1}^{n}\frac{{P}_{n}}{{C}^{i}}\times {S}_{n}$$

$${E}_{r}^{i}={T}_{r}^{i}\times {C}_{f}^{i}$$

where, ${C}^{i}$ is the concentration of contaminant 'i', ${C}_{n}^{i}$ is the abundance of non-contaminated samples (background value), ${T}_{r}^{i}$ is the toxicity coefficient, *P*_*n*_ is the concentration of the specific polymer in MPs, and *S*_*n*_ is the hazard score of MP polymers. The coefficient of toxicity indicates the toxicity level and biological sensitivity (Hakanson, 1980). The five levels of toxicity are: 150 (minor), 150–300 (medium), 300–600 (high), 600–1200 (dangerous), and > 1200 (extreme) (Ranjani et al., 2021).

The Coefficient of Microplastic Impact (CMPI) was assessed to determine the impact of different categories of microplastics, which derive their relationship from shape (Rangel-Buitrago et al., 2021). This coefficient represents the correlation between the total quantity of a particular shape of MPs (e.g., pellets or fibres) and the overall number of MPs identified within a given sampling unit. The CMPI was calculated by using the formula:

$$CMPI= \frac{Specific\; MP\; shape}{Total\; MPs}$$

According to Rangel-Buitrago et al. (2021), the CMPI can be classified into four categories: 0.0001–0.1 (minimum), 0.11–0.5 (average), 0.51–0.8 (maximum), and 0.81–1 (extreme).

### Data Analysis, quality assurance and quality control

The total number of particles obtained in each water category was normalized to particles/L. The number of microplastic particles identified was denoted as the mean (± standard deviation) (SD) of MP particles. The physical attributes of the microplastic particles, such as size, colour, and shape, were presented as relative abundance (%). The normality of the data distribution was evaluated using the Kolmogorov–Smirnov test. The Kruskal–Wallis H test was introduced to identify dissimilarities in the types of MPs found in the sampled locations where the distribution was found to be variable. The significance threshold was set at 0.05. All the data are provided in the supplementary Tables S1–S5. Proper care was taken at all stages of experiments to avoid contamination inside the clean laboratory. Only metal and glass items were used for sample collection and analysis. The workbench was regularly cleansed with ethanol. At the time of conducting the analysis, cotton apron and nitrile gloves were worn. Atmospheric blanks inside the laboratory to check for airborne contamination.

## Results and discussion

### Abundance of microplastics

**Fig. 2:**
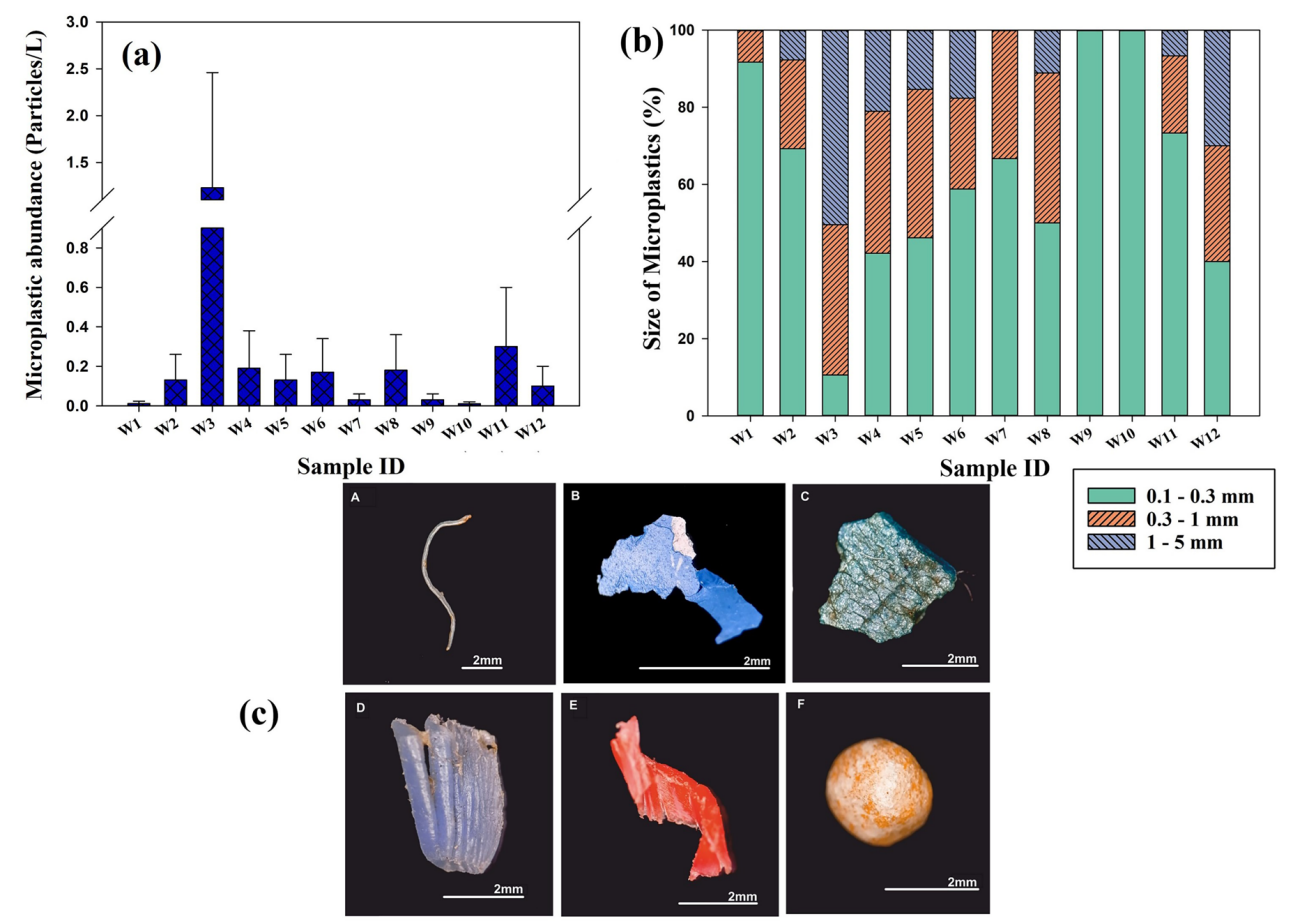
**a** MP abundance in the water samples; **b** percent distribution of MPs across different size intervals in the water samples; and **c** microscopic images of a few visually recognized MPs: fibre (A); fragments (B–E); foam (F)

The laboratory blank samples contained five microplastic fibres, which were reduced in the final dataset. Microplastics were found in all the surface seawater samples collected around the island. The MPs frequency of varied from one place to another (Fig. 2a, Supplementary Table S1). A total of 262 MPs were identified, with an abundance ranging between 1 and 123 particles/100L. The average (± standard deviation) microplastic abundance in surface water for the 12 sampled locations was 0.218 (± 0.329) particles/L. The highest MP abundance (1.23 particles/L) was found in the eastern part of SMI at location W3, with a highly significant difference. An estimated 46.94% of total MPs were identified from this location. The second-highest abundance (0.3 particles/L) was at location W11. The lowest concentration of MPs found was at W10 with 0.01 particles/L. The Kolmogorov–Smirnov test showed that the rate of abundance in surface water (D = 0.37, p = 0.056, Skewness = 3.10, Kurtosis = 10.17) does not differ significantly from that which is normally distributed.

Microplastics are less common on St. Mary's Island than in the South China Sea and the North Atlantic Ocean, where studies have been conducted (Table 1). Nie et al. (2019) found that there were between 1,250 and 3,200 microplastic particles/m^3^ in the South China Sea. This is much more than what was found on St. Mary's Island. Chen et al. (2022) reported an abundance of 4.6 ± 7.0 × 10^7^ items/km^2^ in the Nansha Islands, which is also much higher than the abundance reported on St. Mary's Island. Similarly, the study conducted by Morgana et al. (2018) in the North Atlantic Ocean reported an average microplastic abundance of 2.4 items/m^3^, which is much lower than the abundance reported on St. Mary's Island. Goswami et al. (2020) reported a mean abundance of 0.93 ± 0.59 items/m^3^ in Port Blair Bay, Andaman Island, Bay of Bengal, which is lower than the abundance reported on St. Mary's Island. However, it is important to note that the sampling locations in the two studies are different, and the abundance of microplastics can vary significantly depending on the sampling location. The studies conducted in the Bay of Bengal, such as Jeyasanta et al. (2020) and Patterson et al. (2020), reported microplastic abundances ranging from 24 ± 9 to 126.6 ± 97 items/L, which is relatively higher than the abundance reported on St. Mary's Island. Nahian et al. (2022) conducted a study on Saint Martin Island, Bay of Bengal, and reported an average abundance of 0.118 ± 0.034 items/m^3^, which is much lower than the abundance reported on St. Mary's Island.

**Table 1:** A summary of the basic properties of microplastics reported from water samples of islands in the world oceans

**Reference**	**Study Area**	**Instrumentation**	**Size of MPs**	**Shape of MPs**	**MP abundance**	**Polymer Composition**
Morgana et al. (2018)	Greenland, North Atlantic Ocean	Stereo microscope, ATR-FTIR	0.7 µm—5 mm	Fibre and fragment	2.4 items/ m^3^	PE, PS, PU, PP, EVA, PVC, PA, Polyester
Nie et al. (2019)	Nansha Islands, South China Sea	Stereomicroscope, Micro-Raman spectroscopy	< 0.5 mm, 0.5–1 mm, 1–2 mm, 2–3 mm, 3–5 mm	Fibre, fragment, film, and pellet	1250 to 3200 items/m^3^	PVC, PA, PE, and PP
Ding et al. (2019)	Xisha Islands, South China Sea	Stereozoom microscope, ATR-FTIR, and SEM	20 µm—5 mm	Fibre, fragment, granule, and film	1.0 to 45.2 items/L	Rayon, PET, PA, PE, PTFE, and PVC
Li et al. (2020a, b)	Chongming Island, China	Stereomicroscope, Micro-FTIR	300 µm – 4000 µm	Fibre and fragment	0–259 items/m^3^	PP, PP, α-cellulose, PET, PA, PB, PMMA, cellophane, PU, and EEA
Chen et al. (2022)	Nansha Island, South China Sea	Optical microscope and ATR-FTIR	0.3 – 5 mm	Fibre, fragment, film, and foam	4.6 ± 7.0 × 10^7^ items/km^2^	PE, PP, PS, PET, PA, ABS, PAN, and PMMA
Goswami et al. (2020)	Port Blair Bay, Andaman Island, Bay of Bengal	Microscope and ATR-FTIR	0.05 – 5 mm	Fibre, fragment, and pellet	0.93 ± 0.59 items/m^3^	NY, PU, and PVC
Jeyasanta et al. (2020)	Rameswaram Island, Bay of Bengal	Stereomicroscope, ATR-FTIR, and SEM-EDAX	333 µm – 5 mm	Fibre, fragment, foam, and film	24 ± 9 to 96 ± 57 items/L	PE, PP, PET, PA, PEST, PVC, PS, and PVA
Patterson et al. (2020)	Tuticorin and Vembar Islands, Gulf of Mannar, Bay of Bengal	Stereomicroscope, ATR-FTIR, and SEM-EDAX	100 µm – 5 mm	Fibre, fragment, foam, and film	60 ± 54 to 126.6 ± 97 items/L	PE, PP, PSPA, PEST, PET, PVC, PVA, and PEU
Nahian et al. (2022)	Saint Martin Island, Bay of Bengal	Stereomicroscope, Fourier transform mid-infrared near-infrared (FT-MIR-NIR)	250 – 5000 µm	Fragment, filament, foam, paint flake, and fibre	0.118 ± 0.034 items/m^3^	PP, PS, PET, HDPE, and PA
This study	St. Mary’s Island, Southeastern Arabian Sea	Stereomicroscope, ATR-FTIR, and SEM–EDS	100 µm – 5 mm	Fibre, fragment, film, and foam	0.218 (± 0.329) particles/L	HDPE, PE, PP, LDPE, PS, and PA

### Size, shape, and colour of MPs

The MPs physical characteristics were investigated based on three factors: size, shape, and colour. The size of the extracted microplastics was classified into three ranges: 0.1–0.3 mm, 0.3–1 mm, and 1–5 mm, respectively. The Kruskal–Wallis H test exhibited significance (p < 0.05) between the three size ranges in surface water samples. The highest concentration of MPs in the surface water was found in the size range of 0.1–0.3 mm (37.40%), followed by 0.3–1 mm (32.44%), and 1–5 mm (30.15%) (Fig. 2b, Supplementary Table S1). Identified microplastics at locations W9 and W10 were completely contributed by MPs of 0.1–0.3 mm sizes. The highest abundance of MPs of the three size ranges was found at location W3. Microplastics of size 1–5 mm were dominant (50.41%) at W3. The behaviour, fate, and impacts of microplastics are significantly influenced by particle size (Carbery et al., 2022). Small microplastics possess unique features that distinguish them from plastic debris and larger MPs. They have several properties that make them particularly effective in adsorbing and transporting toxic elements due to their high surface area-to-volume ratio. They can form biofilms that alter their surface charge and ability to clump together with other suspended particles, leading to increased bioavailability in marine ecosystems (Wright et al., 2013). As particle size decreases, MPs become more prevalent and more bio-accessible to marine organisms (Khaleel et al., 2022; Lindeque et al., 2020; Unnikrishnan et al., 2023).

Comparing our data with previously presented studies, the size range of microplastics in this study is between 0.1 mm to 5 mm (Table 1). For example, Morgana et al. (2018) reported the size of microplastics ranging from 0.7 µm to 5 mm in Greenland and the North Atlantic Ocean. Similarly, the study by Ding et al. (2019) in the Xisha Islands, South China Sea, reported microplastics ranging from 20 µm to 5 mm in size. On the other hand, the study by ) on Chongming Island, China, reported microplastics ranging from 300 µm to 4000 µm in size, which overlaps with the size range observed in the St. Mary's Island study.

**Fig. 3:**
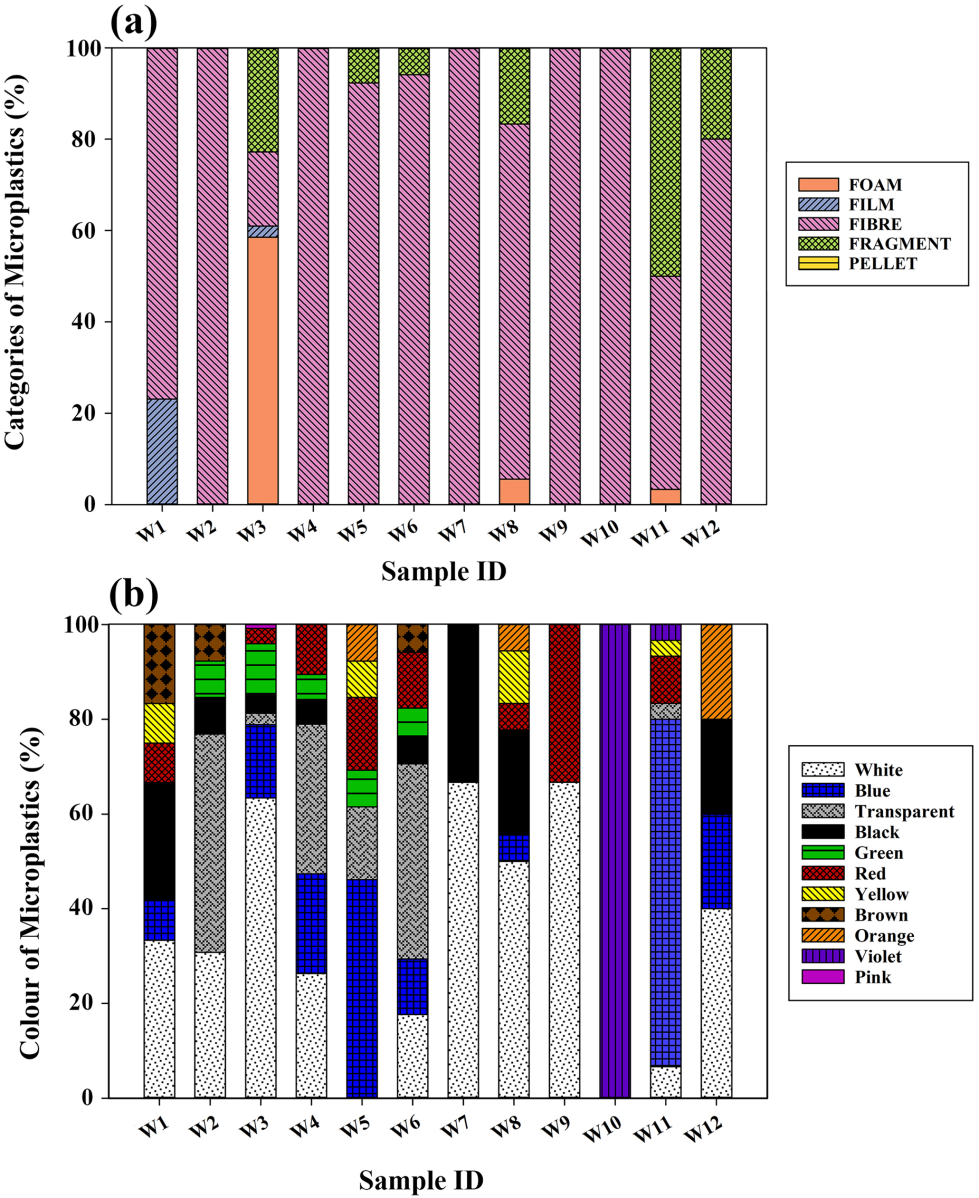
**a** Categories of microplastics present in the water samples; and **b** colour distribution of MPs

Microplastics shapes were classified into five categories: foam, film, fibre, fragment, and pellet. The Kruskal–Wallis H test (p < 0.05) revealed a significant difference in the distribution of various types of MPs in surface water. In the surface water samples, four shapes were identified: foam, film, fibre, and fragment (Fig. 2c). Fibre was the predominant shape, accounting for 50.38%, followed by foam (28.35%), fragment (19.08%), and film (2.29%), respectively (Fig. 3a). Pellets were absent in surface waters. Locations W2, W4, W7, W9, and W10 were completely dominated by fibres. Most of the foams (97.30%), fragments (56%), films (50%), and fibres (15.15%) were identified at location W3.

A wide range of colours, including white, blue, transparent, black, red, green, yellow, brown, orange, violet, and pink, were observed in the water samples collected from St. Mary's Island (Fig. 3b, Supplementary Table S2). The dominant colour observed was white (43.60%), followed by blue (21.93%) and transparent (9.66%). The primary microplastics (MPs) identified in the surface water were white foams and blue fibres. These MPs are primarily derived from the fishing industry located near the island. The Styrofoam packaging and fishing nets used in the industry become abraded and float in the water, making them vulnerable to ingestion by aquatic organisms. This, in turn, increases ecological toxicity in the surrounding environment (Trestrail et al., 2020).

**Fig. 4:**
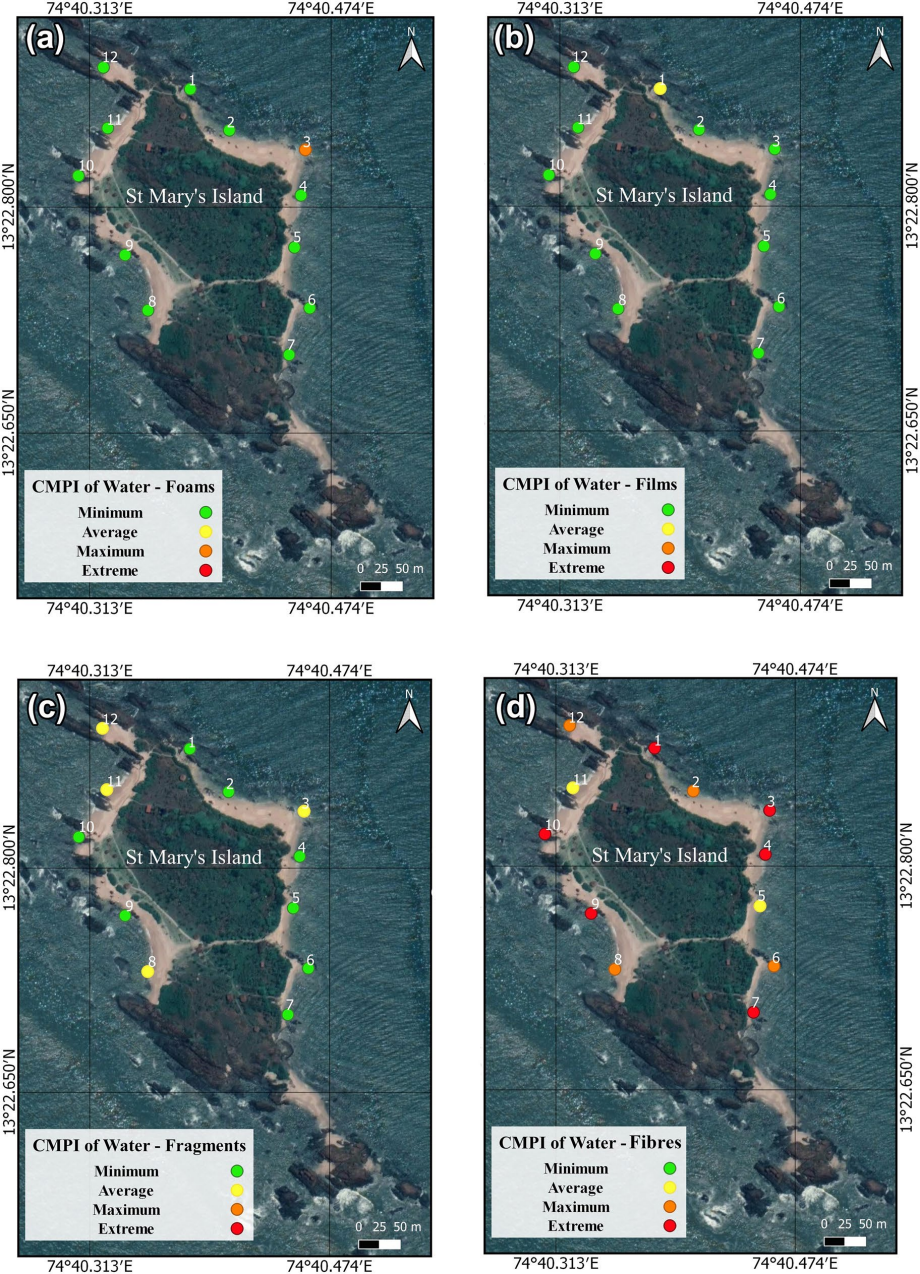
Spatial distribution of the Coefficient Index of Microplastic Impact (CMPI) for **a** foams **b** films **c** fragments and **d** fibres

The Coefficient of Microplastic Impact (CMPI) gives details regarding the environmental effects of a specific MP shape. Here, the CMPI has been calculated for the water samples of St. Mary's Island (Supplementary Table S3). In all four types of shapes that were identified, the coefficient of foam impact was "maximum" (0.51 – 0.8) at site W3 and "minimum" (0.0001 – 0.1) at the other locations (Fig. 4a). The impact of the film was "minimum" at all sampled locations in water except W1, which showed an "average" impact (Fig. 4b). Overall, 66.66% of the locations in water showed a "minimum" impact for fragments, and the rest (W3, W8, W11, and W12) showed an "average" impact (Fig. 4c). There was no "minimum" impact for fibres in water (Fig. 4d). Half of the sampled locations (W1, W3, W4, W7, W9, and W10) in water showed an "extreme" impact of fibres. The "average" impact was on W5 and W11, and the remaining location was categorized under "maximum" impact. All locations registered a "minimum" impact for pellets. The movement of microplastics in the environment is significantly influenced by their shape. Different shapes, such as spherical MPs or elongated fibres, exhibit distinct behaviours, impacting their distribution and accumulation patterns. In the present study, the prevalence and impact of fibres is particularly noteworthy. These fibres, due to their buoyancy, have a higher likelihood of floating in water, posing a considerable risk to aquatic organisms that might inadvertently consume them as food. Additionally, the foams' porous structure makes them highly susceptible to hosting external organic and inorganic agents, providing a conducive environment for the formation and growth of colonies.

### Polymer composition of the MPs

Six types of polymers were identified: low-density polyethylene (LDPE), polystyrene (PS), polyamide (PA), polypropylene (PP), polyethylene (PE), and high-density polyethylene (HDPE). It was found that PS was the most prevalent polymer type in surface water, accounting for 28.24% of the MPs. HDPE, PA, and PE were also identified in significant amounts, accounting for 19.08%, 15.27%, and 11.83%, respectively (Supplementary Table S4). The relative compositions of the different types of polymers are provided in Supplementary Fig. S1, and the FTIR spectra can be found in Khaleel et al. (2022). The results confirm that the primary source of the polymers HDPE and PA is derived from fishing nets. Additionally, the higher quantity of PS indicates significant usage of styrofoam in the storage and transportation of fish from the nearby harbour. The presence of mesoplastics and macroplastics in the samples further supports the inference that LDPE and PE originate from flexible plastic bags and covers.

### Surface morphology and chemical composition of MPs

The surface characteristics of MPs provide insight into the extent of their weathering, which can be determined by observing their morphology under a scanning electron microscope (Zhou et al., 2018). The presence of different surface features on MPs, including scratches, cracks, and pits, offers insights into the extent of their surface abrasion and oxidative reduction. Smooth and clean surfaces are indicative of recently emerged MPs, whereas weathered MPs with signs of abrasion suggest they have been present in the environment for an extended period. This conclusion is supported by relevant morphological evidence (Michael et al., 2015). The roughness of MPs' surfaces can increase microbial accumulation, as demonstrated by Nauendorf et al. (2016).

**Fig. 5:**
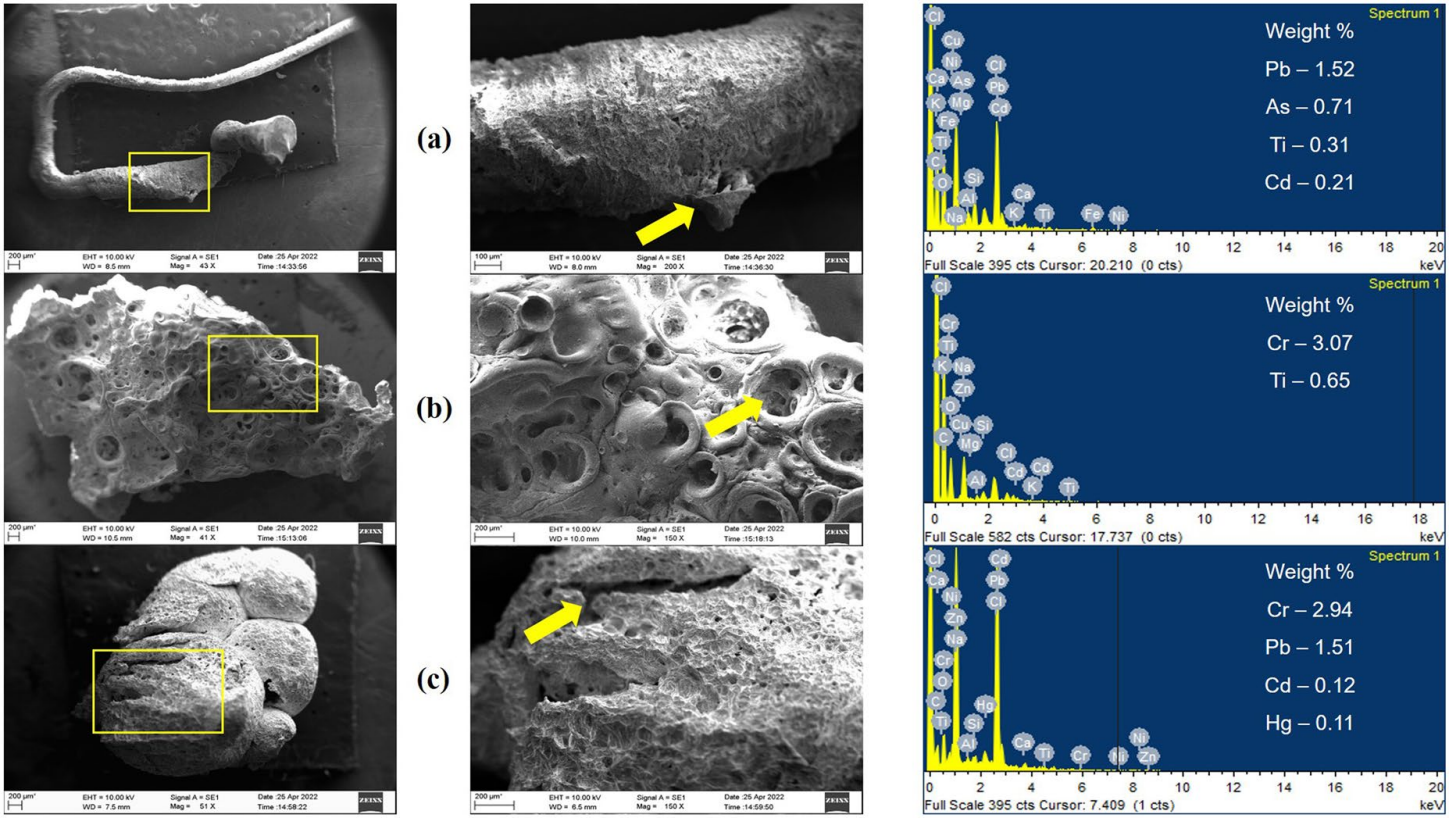
SEM images of microplastics; Fibre (**a**), fragment (**b**), and foam (**c**) and their respective surface elemental compositions

Various heavy metals and inorganic elements, such as aluminum (Al), arsenic (As), cadmium (Cd), chlorine (Cl), chromium (Cr), copper (Cu), iron (Fe), lead (Pb), magnesium (Mg), nickel (Ni), silicon (Si), titanium (Ti), and zinc (Zn), were noticed on the surface of the MPs after their surface composition was examined using an energy-dispersive X-ray spectrometer (Fig. 5). According to Wang et al. (2017), these metals' endogenous origin or the adsorption of pollutants from the nearby marine environment could be the cause of their coexistence with MPs (Brennecke et al., 2016). The ZnCl_2_ solution that was employed during the density separation procedure when isolating MPs is most likely to blame for the elevated concentration of Zn and Cl. It is significant to note that metal contamination from a variety of sources, including metal-based anti-fouling paints, industrial refuse, and fuel combustion, is frequently present in harbours and ports (Deheyn & Latz, 2006). Additionally, inorganic pigments like Fe and Cd are frequently used to colour materials, such as red and yellow (Michael et al., 2015), while chromium is frequently used to stop water corrosion (Oliveira, 2012).

### Risk assessment studies

The polymer hazard index (PHI) values for the identified polymers range from 11.832 (PP) to 847.328 (PS), with a total hazard index of 1933.588, denoting a hazard level of V (> 1000) (Supplementary Table S5). The results indicate that PS and PA have the highest Hazard Index in water, suggesting that their disposal in bodies of water could have significant adverse effects on aquatic life. Conversely, PP has the lowest hazard index, indicating that it poses a relatively lower risk to the environment. According to Yuan et al. (2022), the polymers PS and HDPE can pose a significant threat to human health for those who consume fish that thrive in the ocean waters, which are polluted with these MPs.

The potential ecological risk index (PERI) has been calculated for the water of St. Mary's Island (Supplementary Table S5). The impact is minor (< 150) in three locations. Nearly half of the locations were categorized as medium (150–300). The impact was high (300–600) in two locations (W7 and W9). The average PERI value (203.594) suggests that the island is currently facing a medium environmental risk of MPs. In some specific locations (W7 and W9), the elevated levels of PA and PS led to a more significant PERI value. The island exhibits diversified polymer types with different hazard scores, which indicate a change in pollution levels in the study area.

The highest and lowest MPs concentrations observed were 1.23 particles/L and 0.01 particles/L, respectively, with an average abundance of 0.218 particles/L. The geographical and structural features of the island, as well as hydrological and meteorological conditions, appear to influence this variation (Luo et al., 2019). The northeast side of the island (W3) exhibited the highest MP concentration, which could be attributed to prevailing meteorological conditions during the sampling period. Winds blowing from land towards the sea were recorded by NCEP-NCAR Reanalysis monthly zonal and meridional winds at standard pressure levels on a 2.5° lat/long grid (Khaleel et al., 2022). The distribution of MPs in the north Indian Ocean is significantly influenced by ocean currents, particularly in the vicinity of the island. The transportation and dispersion of MPs from various regions of the Arabian Sea are primarily facilitated by sea surface currents that move toward the island (Khaleel et al., 2022).

The study identified blue fibres and white foams as the primary MPs, followed by blue fragments and fibres of red and black colours. The island receives microplastics from nearby coastal regions and fishing harbours due to its exposure to ocean currents. The island's distinctive hexagonal rhyolitic rock columns, formed from past volcanic activity, play a role in influencing the movement of water and affecting the deposition and distribution of microplastics. These columnar rhyolites create partially enclosed shores, making certain areas hotspots for the accumulation of microplastic particles brought in by waves, particularly notable at location W3. The elevated columnar rhyolites along the island's beaches act as a barrier, leading to the clustering of microplastics in these locations. Main sources of microplastics, identified through collected macroplastics from various sampled spots, include fishing gear and packaging, with the Malpe fishing harbour being a significant contributor in the Arabian Sea. Additionally, rivers near the northeast part of the island are also found to be major pathways for microplastics entering the ocean. Despite being a popular tourist destination, the island's microplastic pollution is not predominantly caused by tourism activities, as strict regulations prohibit plastics within the island perimeter. Instead, the pollution is transported from the shoreline to the island through currents and winds.

### Implications to human health

St. Mary's Island is a habitat for various seaweeds and macroalgae, such as *Phaeophyceae, Rhodophyceae*, and *Chlorophyceae*, as well as a substantial population of Asian green mussels, *Perna viridis (L.)* (Hemachandra & Thippeswamy, 2008; Hemachandra et al., 2017). Previous research has shown that marine organisms, including seaweeds, mussels, and fish, ingest microplastics (Kibria et al., 2022; Kolandhasamy et al., 2018). The size of microplastics appears to be a crucial factor in their ingestion by marine organisms, rather than their shape, with smaller-sized microplastics being more likely to be ingested (Lehtiniemi et al., 2018; Wright et al., 2013). In this study, microplastics with a size range of 0.1–0.3 mm was identified, increasing the bioaccumulation risk. Exposure to microplastics can lead to changes in food habits and various diseases, including endocrine disorders and neoplasia in marine organisms (Ahrendt et al., 2020; Chapron et al., 2018). The study also revealed the presence of harmful elements on the surface of microplastics, which can cause fatal diseases or even death when ingested by organisms. Fishing activities near the island can increase the transfer of microplastics to humans through the food chain. The risk assessment study indicates that while the abundance of microplastics does not pose a significant threat to marine animals, their polymer type and the heavy metals present in them could pose a significant risk. If pollution continues to increase in the future, it may lead to levels that could have adverse consequences for marine biota.

Compared to global islands, the present study suggests that microplastic pollution is of a lower magnitude on St. Mary's Island. To halt or minimize the current production of MPs in the research area, it is essential to address scientific measures such as the provision of incentives for responsible disposal, management, elimination, removal/clean-up strategies, regulatory measures, behavioural change strategies, extended producer liability, source and origin controls, and alteration of conduct (Ogunola et al., 2018; Williams & Rangel-Buitrago, 2019, 2022). The local administration should incorporate techniques (such as beach cleaning exercises and penalizing tourists who pollute the region with plastics) to lessen the convergence of plastics into the marine environment, in addition to measures to alleviate the extended jeopardy of MPs on the island shores. This study provides baseline data for monitoring MP contamination along the island shore, and further investigations on marine biota are expected on a continuous basis to assess the effect of MP ingestion by marine animals.

## Conclusions

This study provides valuable insights into the distribution, composition, and potential ecological risks of microplastic pollution on St. Mary's Island. It was found that the microplastic concentrations in the water samples collected from the island varied, with an average abundance of 0.218 particles/L. The primary microplastics identified were blue fibres and white foams, followed by blue fragments and fibres of red and black colours. The presence of elevated columnar rhyolites along the beaches acted as a barrier, favouring the clustering of microplastics in these locations. Fishing activities and packaging were identified as the main sources of microplastic pollution. The study also revealed the presence of harmful elements on the surface of microplastics, which may pose significant risks to marine organisms.

Based on the Polymer Hazard Index (PHI) and the Potential Ecological Risk Index (PERI), the risk assessment of microplastics showed that the amount of microplastics may not be a big threat to marine animals, but the type of polymer they are made of and the heavy metals they contain could be. The island is currently facing a medium environmental risk from microplastics, and in specific locations, elevated levels of certain polymers led to a higher PERI value. It is crucial to monitor and manage microplastic pollution in the area to mitigate potential adverse effects on marine biota.

To address microplastic pollution on St. Mary's Island, it is vital to implement scientific measures and local administration techniques. These may include incentivizing responsible disposal, management, and elimination of microplastics; implementing regulatory measures and behavioural change strategies; as well as beach cleaning exercises and penalizing tourists who pollute the region with plastics. This study serves as a baseline for monitoring microplastic contamination along the island's shores and emphasizes the need for continuous investigation of marine biota to assess the impact of microplastic ingestion on marine animals.

### Supplementary Information

Below is the link to the electronic supplementary material.

Supplementary file1 (DOCX 416 kb)

## Data Availability

The datasets generated for this study are available from the corresponding author upon request.
