# Supplementary material for: Microplastics in the marine environment of St. Mary's Island: implications for human health and conservation
Source: Environ Monit Assess. 2023 Aug 12;195(9):1034. doi: 10.1007/s10661-023-11651-6 (PMC10421776; doi:10.1007/s10661-023-11651-6)
Supplement: Supplementary file 1 — Supplementary file1 (DOCX 416 kb) [file 10661_2023_11651_MOESM1_ESM.docx]

**Distribution of microplastics and their risks to the marine ecosystem near Saint Mary’s Island, India, Southeast Arabian Sea**

Rizwan Khaleel^a^, Gokul Valsan^b^, Nelson Rangel-Buitrago^c,d^, Anish Kumar Warrier^b,e,*^

^a^Department of Sciences, Manipal Institute of Technology, Manipal Academy of Higher Education, Manipal 576104, Karnataka, India

^b^Department of Civil Engineering, Manipal Institute of Technology, Manipal Academy of Higher Education, Manipal 576104, Karnataka, India.

^c^Programa de Física, Facultad de Ciencias Básicas, Universidad del Atlántico, Puerto Colombia, Atlántico, Colombia.

^d^Programa de Biología, Facultad de Ciencias Básicas, Universidad del Atlántico, Puerto Colombia, Atlántico, Colombia.

^e^Centre for Climate Studies, Manipal Academy of Higher Education, Manipal 576104, Karnataka, India.

Corresponding Author Email address: anish.warrier@manipal.edu

**The file contains supplementary data (tables and figure) related to the manuscript.**

**Supplementary File Table S1. Abundance and size distribution of MPs**

|  | 0.1 - 0.3 mm | | | | | 0.3 - 1 mm | | | | | 1 - 5 mm | | | | |
| --- | --- | --- | --- | --- | --- | --- | --- | --- | --- | --- | --- | --- | --- | --- | --- |
| SAMPLE ID | FOAM | FILM | FIBRE | FRAGMENT | TOTAL | FOAM | FILM | FIBRE | FRAGMENT | TOTAL | FOAM | FILM | FIBRE | FRAGMENT | TOTAL |
| W1 | 0 | 3 | 8 | 0 | 11 | 0 | 0 | 1 | 0 | 1 | 0 | 0 | 0 | 0 | 0 |
| W2 | 0 | 0 | 9 | 0 | 9 | 0 | 0 | 3 | 0 | 3 | 0 | 0 | 1 | 0 | 1 |
| W3 | 1 | 2 | 6 | 4 | 13 | 37 | 0 | 8 | 3 | 48 | 34 | 1 | 6 | 21 | 62 |
| W4 | 0 | 0 | 8 | 0 | 8 | 0 | 0 | 7 | 0 | 7 | 0 | 0 | 4 | 0 | 4 |
| W5 | 0 | 0 | 5 | 1 | 6 | 0 | 0 | 5 | 0 | 5 | 0 | 0 | 2 | 0 | 2 |
| W6 | 0 | 0 | 9 | 1 | 10 | 0 | 0 | 4 | 0 | 4 | 0 | 0 | 3 | 0 | 3 |
| W7 | 0 | 0 | 2 | 0 | 2 | 0 | 0 | 1 | 0 | 1 | 0 | 0 | 0 | 0 | 0 |
| W8 | 0 | 0 | 9 | 0 | 9 | 0 | 0 | 5 | 2 | 7 | 1 | 0 | 0 | 1 | 2 |
| W9 | 0 | 0 | 3 | 0 | 3 | 0 | 0 | 0 | 0 | 0 | 0 | 0 | 0 | 0 | 0 |
| W10 | 0 | 0 | 1 | 0 | 1 | 0 | 0 | 0 | 0 | 0 | 0 | 0 | 0 | 0 | 0 |
| W11 | 0 | 0 | 13 | 9 | 22 | 1 | 0 | 1 | 4 | 6 | 0 | 0 | 0 | 2 | 2 |
| W12 | 0 | 0 | 4 | 0 | 4 | 0 | 0 | 3 | 0 | 3 | 0 | 0 | 1 | 2 | 3 |

**Supplementary File Table S2. Colour of MPs**

| LOCATION | White | Blue | Transparent | Black | Green | Red | Yellow | Brown | Orange | Violet | Pink | Total |
| --- | --- | --- | --- | --- | --- | --- | --- | --- | --- | --- | --- | --- |
| W1 | 4 | 1 | 0 | 3 | 0 | 1 | 1 | 2 | 0 | 0 | 0 | 12 |
| W2 | 4 | 0 | 6 | 1 | 1 | 0 | 0 | 1 | 0 | 0 | 0 | 13 |
| W3 | 78 | 19 | 3 | 5 | 13 | 4 | 0 | 0 | 0 | 0 | 1 | 123 |
| W4 | 5 | 4 | 6 | 1 | 1 | 2 | 0 | 0 | 0 | 0 | 0 | 19 |
| W5 | 0 | 6 | 2 | 0 | 1 | 2 | 1 | 0 | 1 | 0 | 0 | 13 |
| W6 | 3 | 2 | 7 | 1 | 1 | 2 | 0 | 1 | 0 | 0 | 0 | 17 |
| W7 | 2 | 0 | 0 | 1 | 0 | 0 | 0 | 0 | 0 | 0 | 0 | 3 |
| W8 | 9 | 1 | 0 | 4 | 0 | 1 | 2 | 0 | 1 | 0 | 0 | 18 |
| W9 | 2 | 0 | 0 | 0 | 0 | 1 | 0 | 0 | 0 | 0 | 0 | 3 |
| W10 | 0 | 0 | 0 | 0 | 0 | 0 | 0 | 0 | 0 | 1 | 0 | 1 |
| W11 | 2 | 22 | 1 | 0 | 0 | 3 | 1 | 0 | 0 | 1 | 0 | 30 |
| W12 | 4 | 2 | 0 | 2 | 0 | 0 | 0 | 0 | 2 | 0 | 0 | 10 |
| TOTAL | 113 | 57 | 25 | 18 | 17 | 16 | 5 | 4 | 4 | 2 | 1 | 262 |

**Supplementary File Table S3. Risk Assessment Studies: CMPI**

| Sample ID | CMPI-FOAM | CMPI-FOAM Type | CMPI-FILM | CMPI-FILM Type | CMPI-FIBRE | CMPI-FIBRE Type | CMPI-FRAGMENT | CMPI-FRAGMENT Type |
| --- | --- | --- | --- | --- | --- | --- | --- | --- |
| W1 | 0 | MINIMUM | 0.25 | AVERAGE | 0.75 | EXTREME | 0 | MINIMUM |
| W2 | 0 | MINIMUM | 0 | MINIMUM | 1 | MAXIMUM | 0 | MINIMUM |
| W3 | 0.59 | MAXIMUM | 0.02 | MINIMUM | 0.16 | EXTREME | 0.23 | AVERAGE |
| W4 | 0 | MINIMUM | 0 | MINIMUM | 1 | EXTREME | 0 | MINIMUM |
| W5 | 0 | MINIMUM | 0 | MINIMUM | 0.92 | AVERAGE | 0.08 | MINIMUM |
| W6 | 0 | MINIMUM | 0 | MINIMUM | 0.94 | MAXIMUM | 0.06 | MINIMUM |
| W7 | 0 | MINIMUM | 0 | MINIMUM | 1 | EXTREME | 0 | MINIMUM |
| W8 | 0.06 | MINIMUM | 0 | MINIMUM | 0.78 | MAXIMUM | 0.17 | AVERAGE |
| W9 | 0 | MINIMUM | 0 | MINIMUM | 1 | EXTREME | 0 | MINIMUM |
| W10 | 0 | MINIMUM | 0 | MINIMUM | 1 | EXTREME | 0 | MINIMUM |
| W11 | 0.03 | MINIMUM | 0 | MINIMUM | 0.47 | AVERAGE | 0.5 | AVERAGE |
| W12 | 0 | MINIMUM | 0 | MINIMUM | 0.8 | MAXIMUM | 0.2 | AVERAGE |

**Supplementary File Table S4. Polymer composition of MPs**

| LOCATION | HDPE | LDPE | PP | PE | PS | PA | Others | Total |
| --- | --- | --- | --- | --- | --- | --- | --- | --- |
| W1 | 1 | 3 | 0 | 1 | 0 | 5 | 2 | 12 |
| W2 | 3 | 1 | 0 | 0 | 0 | 4 | 5 | 13 |
| W3 | 10 | 7 | 14 | 11 | 72 | 4 | 5 | 123 |
| W4 | 5 | 2 | 0 | 0 | 0 | 5 | 7 | 19 |
| W5 | 7 | 0 | 1 | 1 | 0 | 4 | 0 | 13 |
| W6 | 3 | 1 | 0 | 0 | 0 | 7 | 6 | 17 |
| W7 | 0 | 1 | 0 | 0 | 0 | 2 | 0 | 3 |
| W8 | 7 | 1 | 0 | 4 | 1 | 2 | 3 | 18 |
| W9 | 1 | 0 | 0 | 0 | 0 | 2 | 0 | 3 |
| W10 | 0 | 0 | 0 | 1 | 0 | 0 | 0 | 1 |
| W11 | 9 | 0 | 2 | 13 | 1 | 4 | 1 | 30 |
| W12 | 4 | 1 | 1 | 0 | 0 | 1 | 3 | 10 |
| TOTAL | 50 | 17 | 18 | 31 | 74 | 40 | 32 | 262 |

**Supplementary File Table S5. Results of the risk assessment analysis: PHI and PERI**

| Sample ID | PHI | PHI Type | PERI | PERI Type |
| --- | --- | --- | --- | --- |
| W1 | 2416.67 | Hazard level V | 241.67 | MEDIUM |
| W2 | 1784.62 | Hazard level V | 178.46 | MEDIUM |
| W3 | 2170.73 | Hazard level V | 217.07 | MEDIUM |
| W4 | 1642.11 | Hazard level V | 164.21 | MEDIUM |
| W5 | 2130.77 | Hazard level V | 213.08 | MEDIUM |
| W6 | 2194.12 | Hazard level V | 219.41 | MEDIUM |
| W7 | 3500 | Hazard level V | 350 | HIGH |
| W8 | 1422.22 | Hazard level V | 142.22 | MINOR |
| W9 | 3500 | Hazard level V | 350 | HIGH |
| W10 | 1100 | Hazard level V | 110 | MINOR |
| W11 | 1540 | Hazard level V | 154 | MEDIUM |
| W12 | 1030 | Hazard level V | 103 | MINOR |


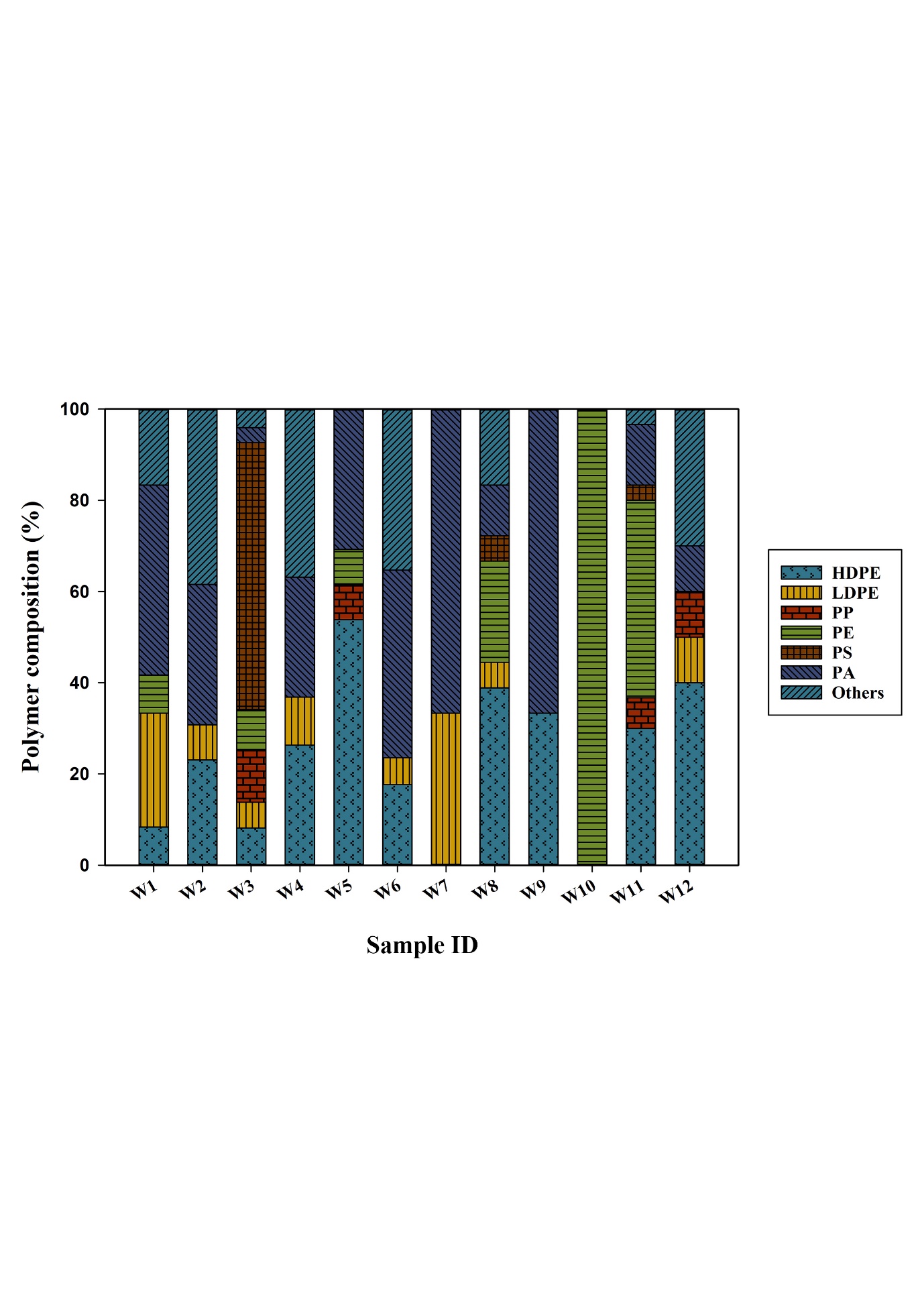


Supplementary Fig. S1. Polymer proportion of MPs
